# Electroacupuncture Reduced Fibromyalgia-Pain-like Behavior through Inactivating Transient Receptor Potential V1 and Interleukin-17 in Intermittent Cold Stress Mice Model

**DOI:** 10.3390/brainsci14090869

**Published:** 2024-08-28

**Authors:** Yu-An Yeh, Hsien-Yin Liao, I-Han Hsiao, Hsin-Cheng Hsu, Yi-Wen Lin

**Affiliations:** 1 Graduate Institute of Acupuncture Science, College of Chinese Medicine, China Medical University, Taichung 404328, Taiwan; 024870@tool.caaumed.org.tw; 2 Department of Chinese Traumatology Medicine, China Medical University Hospital, Taichung 404327, Taiwan; 3 School of Post-Baccalaureate Chinese Medicine, College of Chinese Medicine, China Medical University, Taichung 404328, Taiwan; 017215@tool.caaumed.org.tw (H.-Y.L.); 002762@tool.caaumed.org.tw (H.-C.H.); 4 School of Medicine, College of Medicine, China Medical University, Taichung 404328, Taiwan; 018309@tool.caaumed.org.tw; 5 Department of Traditional Chinese Medicine, China Medical University Hsinchu Hospital, Hsinchu 302056, Taiwan; 6 Chinese Medicine Research Center, China Medical University, Taichung 404328, Taiwan

**Keywords:** fibromyalgia, intermittent cold stress, neuroinflammation, electroacupuncture, TRPV1, IL-17A

## Abstract

Fibromyalgia (FM) is a widespread musculoskeletal pain associated with psychological disturbances, the etiopathogenesis of which is still not clear. One hypothesis implicates inflammatory cytokines in increasing central and peripheral sensitization along with neuroinflammation, leading to an elevation in pro-inflammatory cytokines, e.g., interleukin-17A (IL-17A), enhanced in FM patients and animal models. The intermittent cold stress (ICS)-induced FM-like model in C57BL/6 mice has been developed since 2008 and proved to have features which mimic the clinical pattern in FM patients such as mechanical allodynia, hyperalgesia, and female predominance of pain. Electroacupuncture (EA) is an effective treatment for relieving pain in FM patients, but its mechanism is not totally clear. It was reported as attenuating pain-like behaviors in the ICS mice model through the transient receptor potential vanilloid 1 (TRPV1) pathway. Limited information indicates that TRPV1-positive neurons trigger IL-17A-mediated inflammation. Therefore, we hypothesized that the IL-17A would be inactivated by EA and TRPV1 deletion in the ICS-induced FM-like model in mice. We distributed mice into a control (CON) group, ICS-induced FM model (FM) group, FM model with EA treatment (EA) group, FM model with sham EA treatment (Sham) group, and TRPV1 gene deletion (*Trpv1*^−/−^) group. In the result, ICS-induced mechanical and thermal hyperalgesia increased pro-inflammatory cytokines including IL-6, IL-17, TNFα, and IFNγ in the plasma, as well as TRPV1, IL-17RA, pPI3K, pAkt, pERK, pp38, pJNK, and NF-κB in the somatosensory cortex (SSC) and cerebellum (CB) lobes V, VI, and VII. Moreover, EA and *Trpv1*^−/−^ but not sham EA countered these effects significantly. The molecular mechanism may involve the pro-inflammatory cytokines, including IL-6, IL-17, TNFα, and IFNγ. IL-17A–IL-17RA play a crucial role in peripheral and central sensitization as well as neuroinflammation and cannot be activated without TRPV1 in the ICS mice model. EA alleviated FM-pain-like behaviors, possibly by abolishing the TRPV1- and IL-17A-related pathways. It suggests that EA is an effective and potential therapeutic strategy in FM.

## 1. Introduction

Fibromyalgia (FM) is defined as a chronic syndrome with widespread pain, fatigue, and sleep disturbances [1]. Women have a higher prevalence [2,3], more severe symptoms, and a lower pain threshold than men [4]. The global mean prevalence of FM was reported to be 3.1% and varied with countries, from 0.4% in Greece to 13.4% in Saudi Arabia [5,6]. The mean rate was 3.1% in the Americas, 2.5% in Europe, and 3.3% in Asia. This variation may be because of the differences in the diagnosis methods, the age groups included, and sociocultural beliefs and norms [7].

The etiopathogenesis of FM is not clearly known. Neurotransmitters (neurochemicals) involved in pain processing like serotoninergic, dopaminergic, and opioidergic systems were studied earlier in FM patients. Decreased noradrenalin and increased substance P and glutamate p have been reported. The increased or decreased levels of serotonin, endogenous opioid, opioid receptors, and GABA are still debated. However, their abnormalities were suggested to contribute to the etiology of central sensitization [8,9]. Recently, hypotheses have suggested that inflammatory, peripheral, central, and cognitive–emotional mechanisms interplay to create perceptual impairment of pain [10]. Another one stated that inflammatory cytokines could disrupt the networks of nerves during the interaction of the immune cells with the nervous system. Therefore, it can lead to central and peripheral sensitization along with neuroinflammation [11]. Interleukin (IL)-1β significantly decreased but IL-6, IL-8, interferon γ (IFNγ), tumor necrosis factor-α (TNF-α), C-reactive protein, and brain-derived neurotrophic factor markedly increased in FM patients [12]. These inflammatory biomarkers may not be specific to FM but may play a potential role in FM pathologies. Recently, significantly increased IL-17 levels in the serum and plasma of FM patients [13,14,15] and in intermittent cold stress (ICS)-induced mice [16] were also reported.

IL-17 consists of six members, IL-17A, IL-17B, IL-17C, IL-17D, IL-17E (IL-25), and IL-17F, and is an important pro-inflammatory cytokine [17]. A subset of CD4+ T helper (Th) cells was identified by their expression of IL-17 [18]. Additionally, CD8+ T cells, natural killer T cells, dendritic cells, γδ T cells, macrophages, and other cell types also produce this cytokine and are classified as type 17 cells [19,20]. Under normal physiological conditions, IL-17 supports tissue regeneration, helps maintain mucosal immunity and barrier integrity, and is crucial for overall health and homeostasis [21]. Under pathological conditions, IL-17-related pathways lead to certain immune diseases such as psoriasis, active ulcerative colitis or Crohn’s disease, systemic lupus erythematosus (SLE), multiple sclerosis (MS), and rheumatoid arthritis (RA) and tumors in a wide range of organs including the colon, liver, pancreas, lungs, and bile ducts [22,23,24]. In addition, IL-17 has a crucial role in modulating the immune response involved in neuropathic [25] and inflammatory [26] pains. The IL-17 receptor (IL-17R) family includes five members (IL-17RA–IL-17RE). When IL-17A binds to IL-17R, it stimulates pro-inflammatory signaling pathways such as the phosphoinositide 3-kinase/protein kinase B (PI3K/AKT), mitogen-activated protein kinases (MAPKs), and nuclear factor-κB (NF-κB)-mediated pathways [27,28,29].

The PI3K/Akt pathway regulates cell regeneration, apoptosis, and cognitive memory in the central nervous system (CNS) [30,31,32], as well as inflammation, cell death, and glial scar formation after spinal cord injury [33]. It also plays a critical role in neuropathic and inflammatory pains [34,35]. MAPKs are crucial for intracellular signal transduction involved in modulating neuropathic pain and inflammatory responses. The MAPK family includes three major members. They are extracellular signal-regulated kinase (ERK), p38, and c-Jun N-terminal kinase (JNK). MAPK pathways regulate the hypersensitivity to pain, inflammatory pain, and neuropathic pain in the neurons [36]. Phosphorylated extracellular signal-regulated kinase (pERK) is involved in FM-associated pain in both the peripheral nervous system (PNS) and CNS [37,38]. The p38–MAPK-activated protein kinases 2 (MK2) axis may be a pathological mechanism in FM patients [39]. Nuclear factor-κB (NF-κB) regulates the transcription of genes involved in the inflammatory response processes [40,41] and regulates CNS-associated pain in FM-like pain models [42,43].

Recently, it was found that the activation of the cutaneous transient-receptor-potential-vanilloid-1-positive (TRPV1+) nerves alone is sufficient to trigger local inflammation, which is specific to type 17 cells and dependent on IL-17A [44]. TRPV1, a non-selective transmembrane cation channel protein, can be activated by either physical or chemical stimuli. It is involved in the development and maintenance of nociception in an FM model [38,45]. It plays a vital role in pain induction in the peripheral tissues and the spinal cord [46]. It was also proved to be involved in nociceptive processing in the brain [47,48,49,50,51,52,53]. It is expressed in the cerebral cortex, cerebellum, hippocampus, thalamus, substantia nigra, and central amygdala in rodents [54,55].

Cumulating evidence showed that non-pharmaceutical therapy, including therapeutic massage, exercise, dry needling, and electrotherapy (such as transcutaneous electrical nerve stimulation, transcranial magnetic stimulation, transcranial direct current, and laser), has been used in treating FM patients [9,56]. Acupuncture, the practice of inserting needles into the skin and deeper tissues, based on traditional Chinese medicine theory since 2600 BC, has been widely used [57]. Electroacupuncture (EA) is a mode of treatment that combines manual acupuncture and electrostimulation. Regarding FM therapies, acupuncture and EA are effective and safe treatments for relieving pain and associated symptoms, as well as improving the quality of life in FM patients [58,59,60,61,62,63]. The expression of TRPV1 was enhanced in FM models and attenuated by EA through TRPV1 and its related downstream signaling pathway molecules in the dorsal root ganglion (DRG), somatosensory cortex (SSC), thalamus, prefrontal cortex (PFC), hippocampus, periaqueductal gray (PAG), amygdala, and cerebellum (CB) of mice brains [16,64,65,66,67,68]. EA alleviates the inflammatory reaction by reducing the levels of serum pro-inflammatory cytokines such as IL-1, IL-1β, IL-6, IL-17, IL-23, and TNF-α in rheumatoid arthritis (RA) animal models [69] and IL-17 in the peripheral blood of RA patients [70]. A previous study revealed that EA reduced the pro-inflammatory cytokines and suppressed the TRPV1-related pathways in the plasma and brain in an ICS-induced FM model mice [71]. The ICS-induced FM model mice share similarities in pathophysiology (such as female-predominant, widespread, and long-lasting pain) and pharmacotherapeutic properties (such as positive effects with pregabalin and serotonin and norepinephrine reuptake inhibitors [SNRIs] and negative effects with non-steroidal anti-inflammatory drugs [NSAIDs] and morphine) which mimic FM patients [72].

After the previous review, it was known that TRPV1- and IL-17A-mediated pathways play a critical role in inflammation and pain in FM. IL-17A and inflammation could be triggered by a TRPV1-positive neuron when it received stimuli. We have reported that EA alleviated pain-like response through TRPV1 in ICS-induced FM model mice [16]. Therefore, we hypothesized that the IL-17A would be inactivated by EA and TRPV1 deletion. It is the first study to investigate IL-17A and its mediated molecules’ response to EA as well as TRPV1 deletion in the ICS-induced experimental FM mice model.

## 2. Materials and Methods

### 2.1. Experimental Animals and Ethical Considerations

There were 40 C57BL/6 female mice aged 8–12 weeks and weighing 18–20 g in this study in total. A total of 32 female wild-type (WT) mice (BioLASCO Taiwan Co., Ltd., Taipei, Taiwan) and 8 female TRPV1-knockout (*Trpv1*^−/−^) mice (Jackson Lab., Bar Harbor, ME, USA) were used. All mice were treated according to the National Institute of Health’s Guide for the Care and Use of Laboratory Animals. The experimental protocol (CMUIACUC-2021-343) was approved by the Institutional Animal Care and Use Committee of China Medical University. A sample size of 8 mice per group was estimated and calculated by G*power software version 3.1.9.7 to minimize the number of mice required for reliable data. We chose the f-test, repeated measures, and within–between interaction for ANOVA. The input parameters were effect size (EZ) = 0.4 (medium), alpha = 0.05, power (1 − *β*) = 0.8, number of groups = 5, and correlation among repeated measures = 0.5. The estimated total sample size was 40 (8 mice per group). The undue suffering during the study was minimized. Mice were anesthetized with 1% isoflurane before acupuncture and cervical dislocation to avoid them experiencing pain distress. Mice were given optimal care in Plexiglas cages and kept in a room with a 12:12 h light–dark cycle (8:00 a.m. to 8:00 p.m.) maintained at a temperature of 24 ± 1 °C and relative humidity of 60 ± 5%. All WT mice were randomly subdivided into four groups: Control group (CON group), ICS-induced FM model group (FM group), FM model receiving electroacupuncture group (EA group), and FM model receiving sham electroacupuncture group (Sham group). The fifth group was the *Trpv1*^−/−^ mice group (*Trpv1*^−/−^ group).

### 2.2. The Intermittent Cold Stress (ICS)-Induced Fibromyalgia (FM)-like Mice Model

The ICS-induced FM-like mice model was developed by Michiko Nishiyori and Hiroshi Ueda [73]. The model was proved to be a good model that mimics fibromyalgia pain syndromes in humans including mechanical allodynia, hyperalgesia, and female predominance of pain. All mice were kept at room temperature (24 ± 1 °C) before the experiments. For the ICS treatment, two mice were housed in a Plexiglas cage (13 × 18.8 × 29.5 cm) covered with a stainless-steel mesh. The mice were then placed in a cold room at 4 °C overnight, from 16:30 to 10:00 (Day 0 to Day 1). At 10:00 on Day 1, the mice were moved to 24 °C for 30 min and moved back to 4 °C for 30 min. The process was repeated to subject the mice to changes in environment temperature (24 °C and 4 °C) intermittently between 10:00 and 16:30. After that, the mice were placed in the 4 °C cold room overnight from 16:30 to 10:00 (from Day 1 to 2). The mice received intermittent temperature changes (24 °C and 4 °C) between 10:00 and 16:30 on Day 2 again. Finally, the mice were placed in the 4 °C cold room overnight from 16:30 and were moved out to 24 °C at 10:00 on Day 3. Except for the CON group, which was kept at room temperature with no interventions throughout the experiment, the groups received ICS treatment.

### 2.3. Electroacupuncture (EA) and Sham EA Treatments

We applied the acupuncture to EA and sham EA groups after mice received ICS treatment. After mice were anesthetized with 1% isoflurane, we inserted the stainless-steel needles (diameter, 0.23 mm; length, 13 mm; Shanghai Yang Long Medical Articals Co., Ltd., Shanghai, China) into the bilateral Zusanli (ST36) acupoint to a depth of 3 mm. The ST36 point is located approximately 4 mm below and 1–2 mm lateral to the midpoint of the knee on the hind limb of mice [74]. In the EA group, electrical stimuli were administered using a Trio 300 stimulator (Ito Co., Ltd., Saitama, Japan) at an intensity of 1 mA for 20 min at a frequency of 2 Hz and a pulse width of 100 μs. The frequency (Hz), duration, and intensity of EA followed the previous protocol we published before [16]. The EA treatment caused slight, visible muscle twitching around the insertion area. The retention of the needle at ST36 in sham EA group mice without electrical stimuli was also maintained for 20 min. The EA stimulation and sham EA needling were applied once a day from Day 4 to Day 8. The acupuncture practitioner is a licensed Chinese Medicine doctor in Taiwan who has performed acupuncture on humans in clinic since 2015 and has participated in animal experimentation since 2019.

### 2.4. Behavior Test

The mechanical and thermal pain-like behavior was tested at Day 0 as a pre-test before the ICS induction and tested at Day 8 as a post-test before the sacrifice. Prior to the behavior tests, all mice were transported to the behavior analysis room and allowed to acclimate to the environment for at least 30 min. All experiments were conducted at room temperature (24 ± 1 °C), and stimuli were applied only when the mice were calm but not sleeping or grooming. The von Frey filament test was conducted first. The mice were placed on a metal mesh (75 × 25 × 45 cm) and covered with a Plexiglas cage (10 × 6 × 11 cm) to acclimate for a minimum of 30 min. Mechanical sensitivity was assessed by measuring the force of responses to stimulation with three applications of the electronic, calibrated von Frey filament (IITC Life Science Inc., Woodland Hills, CA, USA). The mice were then mechanically stimulated at the plantar region of the right hind paw using the tip of the filament. The filament gram counts were documented when the stimulation prompted the mouse to withdraw its hind paw. Secondly, the Hargreaves’ assessment was employed to measure thermal pain-like behavior by testing the response time to thermal stimulation with three applications using the Hargreaves’ test IITC analgesiometer (SERIES8; Model 390G; IITC Life Sciences Inc., Woodland Hills, CA, USA). The mice were placed in a Plexiglas cage on top of a glass sheet and allowed to acclimate for at least 30 min. The thermal stimulator was placed beneath the glass sheet with the projection bulb focused precisely on the middle of the plantar surface of the right hind paw. A cut-off time of 20 s was established to prevent tissue damage. During the thermal paw withdrawal test, the nociception threshold was determined by recording the latency of paw withdrawal when the constant heat stimulation caused the mouse to withdraw its hind paw.

### 2.5. Enzyme-Linked Immunosorbent Assay (ELISA) and Western Blot Analysis

After all experimental treatment, at day 8, the mice were anesthetized with 1% isoflurane and underwent cervical dislocation. The mice plasma was collected by retro-orbital sinus puncture and evaluated through Q-Plex™ Mouse Cytokine Screen quantitative ELISA-based chemiluminescent assay (Quansys Biosciences, Logan, UT, USA). The tissues of the somatosensory cortex (SSC) and cerebellum lobules V, VI, and VII (CB5, CB6, CB7) were immediately excised for protein extraction, initially placed on ice, and then stored at −80 °C until protein extraction. Total proteins were homogenized in cold radioimmunoprecipitation (RIPA) lysis buffer containing 50 mM Tris-HCl (pH 7.4), 250 mM NaCl, 1% NP-40, 5 mM EDTA, 50 mM NaF, 1 mM Na_3_VO_4_, 0.02% NaN_3_, and 1× protease inhibitor cocktail (AMRESCO). The extracted proteins underwent 8% SDS-Tris glycine gel electrophoresis and were subsequently transferred to a PVDF membrane. After blocking the membrane with 5% non-fat milk in TBS-T buffer (10 mM Tris, pH 7.5, 100 mM NaCl, 0.1% Tween 20), the membrane was incubated for one hour at room temperature with a primary antibody against IL-17RA (~130 kDa, 1:1000, cat #: ab180904, Abcam, Cambridge, UK), pPI3K (~125 kDa, 1:1000, cat #: PA5-28070, Invitrogen, Waltham, MA, USA), TRPV1 (~95 kDa, 1:1000, cat #: ACC-030, Alomone Labs Ltd., Jerusalem, Israel), pNF*κ*B (~65 kDa, 1:1000, cat #: ab86299, Abcam, Cambridge, UK), pAkt (~60 kDa, 1:1000, cat #: 9271, Cell Signaling Technology, Danvers, MA, USA), pERK1/2 (~42–44 kDa, 1:1000, cat #: 36-8800, Invitrogen, MA, USA), pp38 (~41 kDa, 1:1000, cat #: 44-684G, Invitrogen, MA, USA), and pJNK (~42 kDa, 1:1000, cat #: 44-682G, Invitrogen, MA, USA) in TBS-T containing 1% bovine serum albumin (BSA). The suitable secondary antibody was either peroxidase-conjugated anti-rabbit, anti-mouse, or anti-goat antibody (1:5000). The bands were visualized using the LAS-3000 Fujifilm (Fuji Photo Film Co., Ltd., Tokyo, Japan) and an enhanced chemiluminescent substrate kit (Pierce™, Waltham, MA, USA). When necessary, NIH ImageJ software version 1.53e (Bethesda, MD, USA) was utilized to quantify the intensities of specific bands. The internal control used was α-tubulin.

### 2.6. Immunofluorescence

The mice were euthanized by inhalation of 5% isoflurane, then received an intracardial perfusion of normal saline and 4% paraformaldehyde. The brain was dissected right away, and it was post-fixed for three days at 4 °C using 4% paraformaldehyde. The tissues were cryoprotected for a whole night at 4 °C in 30% sucrose. The brain was embedded in an OCT compound and immediately frozen using liquid nitrogen, and the tissues were stored at −80 °C. Frozen segments were immediately placed on glass slides after being cut at a 20 μm width on a cryostat. After the samples were fixed with 4% paraformaldehyde, they were incubated for one hour at room temperature with the blocking solution Normal goat serum (#005-000-121; Jackson ImmunoResearch©, St. Thomas’ Place, Ely, UK). The samples were incubated at 4 °C overnight with the primary antibodies pERK (1:200, cat #: 36-8800, Invitrogen, MA, USA) and IL-17RA (1:200, cat #: ab180904, abcam, Cambridge, UK) after blocking. The samples were then fixed with cover slips for immunofluorescence visualization after being incubated for two hours at room temperature with the secondary antibody (1:500), 488-conjugated AffiniPure donkey anti-rabbit IgG (H + L), 594-conjugated AffiniPure donkey anti-goat IgG (H + L), and Peroxidase-conjugated AffiniPure donkey anti-mouse IgG (H + L). To detect nonspecific binding from the secondary antibody, a negative control was also added. An epi-fluorescent microscope (Olympus, BX-51, Tokyo, Japan) with a 20× numerical aperture (NA = 0.4) objective was used to observe the samples. NIH ImageJ software version 1.53e (Bethesda, MD, USA) was used to analyze the images.

### 2.7. Statistical Analysis

SPSS version 12 was used for the statistical evaluation. The mean and standard error are presented for all statistical data. To assess the normality of the results, the Shapiro–Wilk test was performed. A post hoc Tukey’s test and repeated-measures ANOVA were used to assess statistical significance among all groups. A *p*-value of 0.05 was considered statistically significant.

## 3. Results

**Figure 1:**
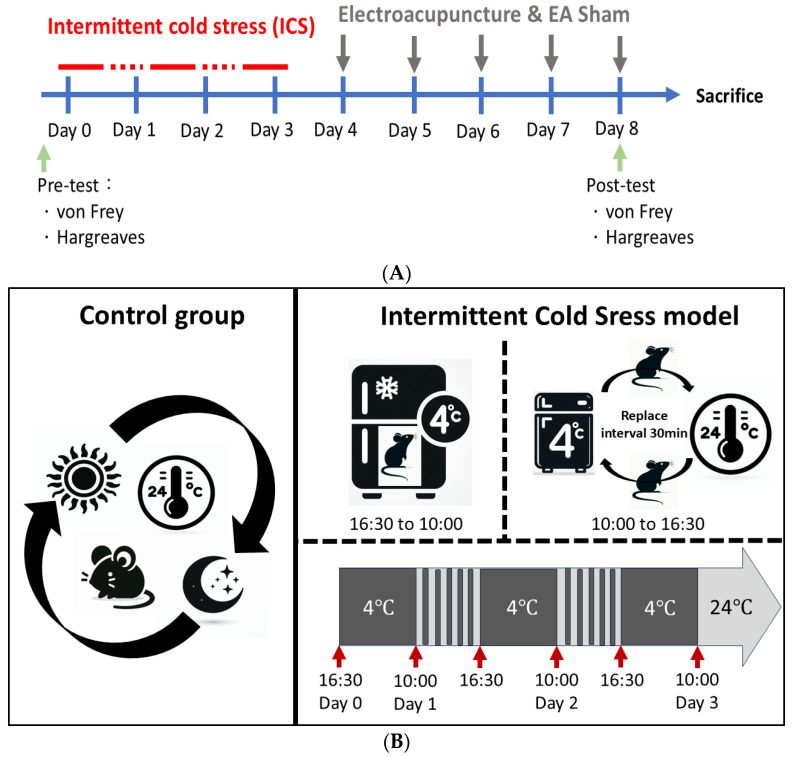
(**A**) All mice underwent von Frey test for mechano-sensation and Hargreaves’ test for thermal nociception before any treatment on Day 0 (pre-test). CON group did not receive any treatment throughout the study. FM group, EA group, Sham group, and *Trpv1*^−/−^ group received ICS treatment from Day 0 to Day 3 for FM model development (**B**). Then, EA and sham EA were given once a day from Day 4 to Day 8. EA group received 2 Hz and 1 mA EA at bilateral ST36 for 20 min each time. Sham group received acupuncture without electric stimulation at bilateral ST36 for 20 min each time. After that, all groups received a post-test. Finally, all mice were sacrificed, and their plasma and brain tissue were collected and analyzed. The diagram (**B**) illustrates the ICS protocol. To develop the FM model, mice were first kept at 4 °C for 17.5 h from 16:30 to 10:00 (from Day 0 to 1). Then, the mice were moved to 24 °C for 30 min and then moved back to 4 °C for 30 min. From 10:00 to 16:30 on Day 1, the mice were moved between 24 °C and 4 °C for 30 min each time. The procedure was repeated and terminated on Day 3 at 10:00. CON = control. FM = intermittent cold stress (ICS)-induced FM-like mice model. EA = electroacupuncture. Sham = sham EA. *Trpv1*^−/−^ = transient receptor potential vanilloid 1 gene knock out.

Figure 1 illustrates the study design and ICS protocol.

### 3.1. Effect of Electroacupuncture Treatment and TRPV1 Deletion on Pain-like Behavior and the Levels of Inflammatory Mediators in Fibromyalgia Model Mice

**Figure 2:**
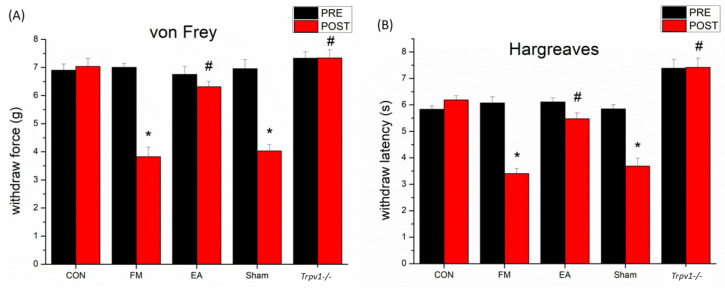
Intermittent-cold-stress-induced fibromyalgia mice model (FM) had (**A**) mechanical hyperalgesia in von Frey test and (**B**) thermal hyperalgesia in Hargreaves’ test (* *p* < 0.05, n = 8). PRE = pre-test. POST = post-test. CON = control. FM= intermittent cold stress (ICS)-induced FM-like mice model. EA = electroacupuncture. Sham = sham EA. *Trpv1*^−/−^ = transient receptor potential vanilloid 1 gene knock out. Asterisks (*) denote statistical significance compared to the control group, while hashtag symbols (#) indicate statistical significance compared to the FM group.

The von Frey test and Hargreaves’ method were used to evaluate mechanical allodynia and thermal hyperalgesia in mice. In the beginning, mechanical and thermal nociceptive responses showed no significant differences among the five groups without treatment (Figure 2A,B). The control group (without any intervention) did not represent any marked variation in mechanical and thermal pain-like sensations (7.04 ± 0.28 g, n = 8; 6.19 ± 0.16 s, n = 8) throughout the experiment. After ICS treatment, typical mechanical allodynia and thermal hyperalgesia were induced, and the mechanical threshold (g) and thermal latency (s) were remarkably lower in the FM group (3.83 ± 0.33 g, n = 8, * *p* < 0.05; 3.41 ± 0.19 s, n = 8, * *p* < 0.05). Asterisks (*) indicate statistical significance when compared with the control group. Regarding the EA group, the FM model received EA at ST36, and mechanical allodynia and thermal hyperalgesia were both significantly diminished when compared to the FM group (6.32 ± 0.19 g, n = 8, # *p* < 0.05; 5.48 ± 2.22 s, n = 8, # *p* < 0.05). Hashtag symbols (#) indicate statistical significance when compared with the FM group. However, these phenomena were not observed in the Sham group (4.03 ± 0.023 g, n = 8, * *p* < 0.05; 3.69 ± 0.29 s, n = 8, * *p* < 0.05). Mechanical allodynia and thermal hyperalgesia were also reversed in the *Trpv1*^−/−^ group when compared to the FM group (7.34 ± 0.28 g, n = 8, # *p* < 0.05; 7.42 ± 0.35 s, n = 8, # *p* < 0.05).

**Figure 3:**
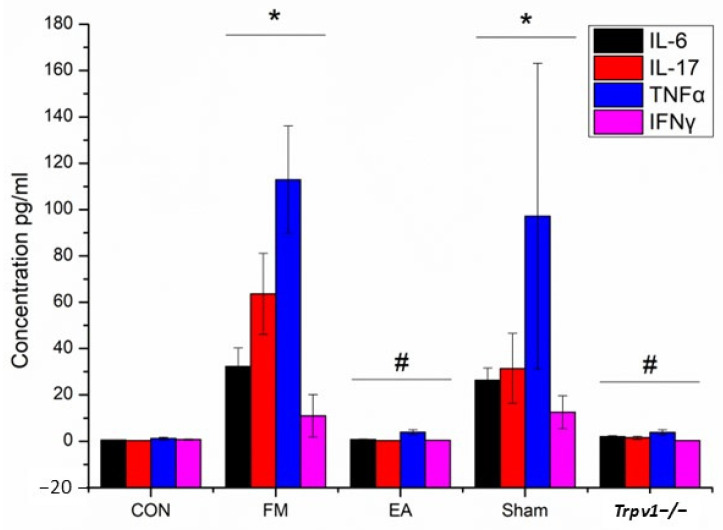
Multiplex ELISA test showed the increased level of IL-6, IL-17, TNFα, and IFNγ in mice plasma (* *p* < 0.05, n = 5). EA and TRPV1 deletion reversed these effects (# *p* < 0.05). CON = control. FM = intermittent cold stress (ICS)-induced FM-like mice model. EA = electroacupuncture. Sham = sham EA. *Trpv1*^−/−^ = transient receptor potential vanilloid 1 gene knock out. IL-6 = interleukin-6. IL-17 = interleukin-17. TNF-α = tumor necrosis factor-α. IFNγ = interferon γ. Asterisks (*) denote statistical significance compared to the control group, while hashtag symbols (#) indicate statistical significance compared to the FM group.

The concentration of pro-inflammatory mediators in mouse plasma was also evaluated (Figure 3). These mediators in the FM group (n = 5, IL-6: 32.3 ± 7.9 pg/mL; IL-17: 63.6 ± 17.4 pg/mL; TNFα: 112.9 ± 23.1 pg/mL; and IFNγ: 11.0 ± 9.2 pg/mL) and the Sham group (n = 5, IL-6: 26.3 ± 5.3 pg/mL; IL-17: 31.4 ± 15.1 pg/mL; TNFα: 97.2 ± 66.0 pg/mL; and IFNγ: 12.5 ± 7.1 pg/mL) increased significantly (* *p* < 0.05) when compared to the CON group (n = 5, IL-6: 0.5 ± 0.2 pg/mL; IL-17: 0.3 ± 0.1 pg/mL; TNF-α: 1.1 ± 0.6 pg/mL; and IFNγ: 0.7 ± 0.2 pg/mL). However, these mediators in the EA group (n = 5, IL-6: 0.8 ± 0.2 pg/mL; IL-17: 0.3 ± 0.1 pg/mL; TNFα: 3.8 ± 1.1 pg/mL; and IFNγ: 0.5 ± 0.1 pg/mL) and *Trpv1*^−/−^ group (n = 5, IL-6: 2.0 ± 0.4 pg/mL; IL-17: 1.5 ± 0.7 pg/mL; TNFα: 3.7 ± 1.3 pg/mL; and IFNγ: 0.3 ± 0.1 pg/mL) were markedly lower (# *p* < 0.05) than in the FM group.

### 3.2. Electroacupuncture (EA) and TRPV1 Deletion (Trpv1^−/−^) but Not Sham EA Reduced IL-17-Related Signaling Pathways in the Somatosensory Cortex (SSC) of Mice 

**Figure 4:**
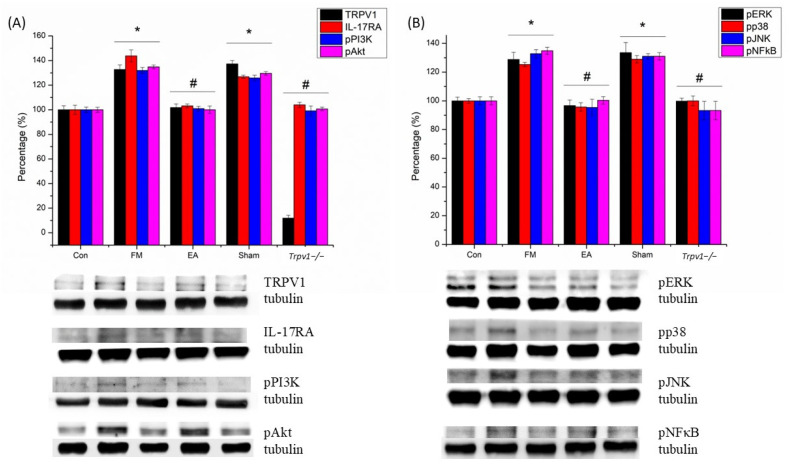
(**A**,**B**) Western blot showed that ICS increased the level of TRPV1, IL-17RA, pPI3K, pAkt, pERK, pp38, pJNK, and pNF-κB in the SSC of mice. SSC = somatosensory cortex. CON = control. FM = intermittent cold stress (ICS)-induced FM-like mice model. EA = electroacupuncture. Sham = sham EA. *Trpv1*^−/−^ = transient receptor potential vanilloid 1 gene knock out. TRPV1 = transient receptor potential vanilloid 1. IL-17RA = interleukin-17 receptor A. pPI3K = phosphorylated phosphoinositide 3-kinase. pAkt = phosphorylated protein kinase B. pERK = phosphorylated extracellular signal-regulated kinase. pp38 = phosphorylated p38. pJNK = phosphorylated c-Jun N-terminal kinase. pNF-κB = phosphorylated nuclear factor-κB. Asterisks (*) denote statistical significance compared to the control group, while hashtag symbols (#) indicate statistical significance compared to the FM group.

**Figure 5:**
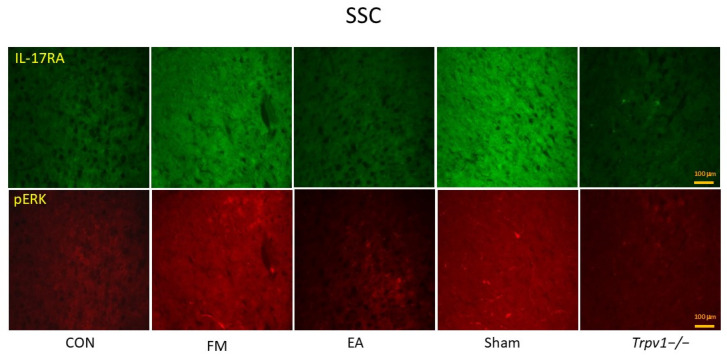
Immunofluorescence staining (n = 2) showed the increased signals of IL-17RA and pERK in FM groups. However, the EA and TRPV1 deletion reversed the effects. SSC = somatosensory cortex. CON = control. FM= intermittent cold stress (ICS)-induced FM-like mice model. EA = electroacupuncture. Sham = sham EA. *Trpv1*^−/−^ = transient receptor potential vanilloid 1 gene knock out. IL-17RA = interleukin-17 receptor A. pERK = phosphorylated extracellular signal-regulated kinase.

We used Western blots to investigate the levels of TRPV1, IL-17, and their related downstream proteins PI3K, AKT, JNK, ERK, p38, and NF-kB in the SSC of mice. The results (Figure 4A,B) showed that the amounts of phosphorylated IL-17 (pIL-17), pPI3K, pAKT, pJNK, pERK, pp38, and pNF-kB were significantly up-regulated after ICS induction in FM and Sham groups when compared to the CON group (n = 6, * *p* < 0.05). However, EA and *Trpv1*^−/−^ considerably attenuated this effect (n = 6, # *p* < 0.05). We also performed immunofluorescence staining of the mouse SSC. The IL-17- and ERK-associated signals were low in normal mice but augmented in FM mice (n = 2; Figure 5). The increased signals were alleviated by EA and *Trpv1*^−/−^ but not sham EA.

### 3.3. Electroacupuncture (EA) and TRPV1 Deletion (Trpv1^−/−^) but Not Sham EA Reduced IL-17-Related Signaling Pathways in the Cerebellum Lobe V (CB5) of Mice

**Figure 6:**
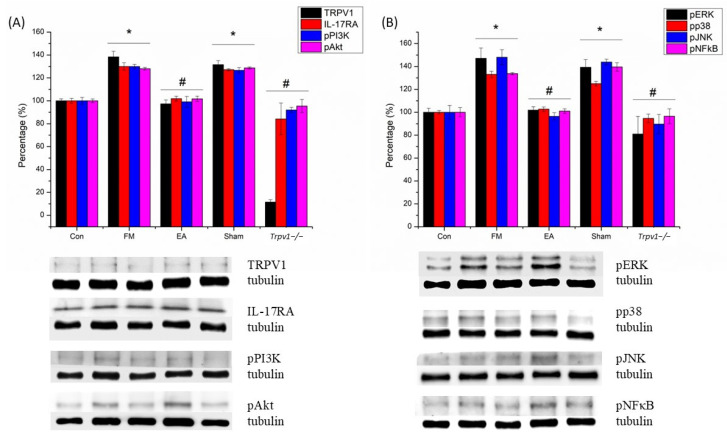
(**A**,**B**) Western blot showed that ICS increased the level of TRPV1, IL-17RA, pPI3K, pAkt, pERK, pp38, pJNK, and pNF-κB in the CB5 of mice. CB5 = cerebellum lobe V. CON = control. FM = intermittent cold stress (ICS)-induced FM-like mice model. EA = electroacupuncture. Sham = sham EA. *Trpv1*^−/−^ = transient receptor potential vanilloid 1 gene knock out. TRPV1 = transient receptor potential vanilloid 1. IL-17RA = interleukin-17 receptor A. pPI3K = phosphorylated phosphoinositide 3-kinase. pAkt = phosphorylated protein kinase B. pERK = phosphorylated extracellular signal-regulated kinase. pp38 = phosphorylated p38. pJNK = phosphorylated c-Jun N-terminal kinase. pNF-κB = phosphorylated nuclear factor-κB. Asterisks (*) denote statistical significance compared to the control group, while hashtag symbols (#) indicate statistical significance compared to the FM group.

**Figure 7:**
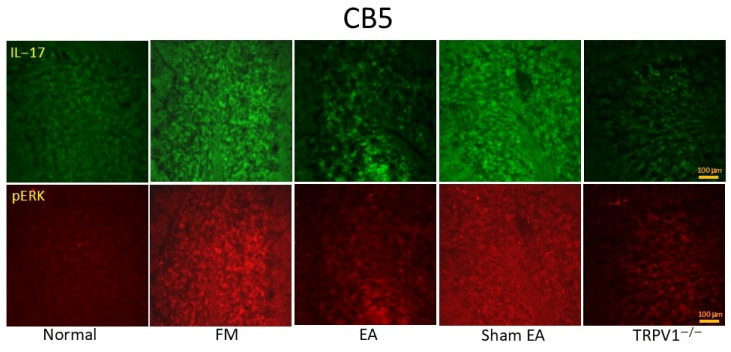
Immunofluorescence staining (n = 2) showing the increased signals of IL-17RA and pERK in the FM groups. EA and TRPV1 deletion could reverse these effects. CB5 = cerebellum lobe V. CON = control. FM = intermittent cold stress (ICS)-induced FM-like mice model. EA = electroacupuncture. Sham = sham EA. *Trpv1^−^^/^^−^* = transient receptor potential vanilloid 1 gene knock out. IL-17RA = interleukin-17 receptor A. pERK = phosphorylated extracellular signal-regulated kinase.

In this study, pTRPV1, pIL-17, pPI3K, pAKT, pJNK, pERK, pp38, and pNF-kB were increased in the CB5 of FM and Sham group mice (Figure 6; n = 6, * *p* < 0.05). EA and *Trpv1*^−/−^ also markedly suppressed these proteins in CB5 (n = 6, # *p* < 0.05). Immunofluorescence indicated that IL1-7 and ERK were elevated in the CB5 of FM mice, which was reversed by EA but not sham EA (Figure 7; n = 6).

### 3.4. Electroacupuncture (EA) and TRPV1 Deletion (Trpv1^−/−^) but Not Sham EA Reduced IL-17-Related Signaling Pathways in the Cerebellum Lobe VI (CB6) of Mice

**Figure 8:**
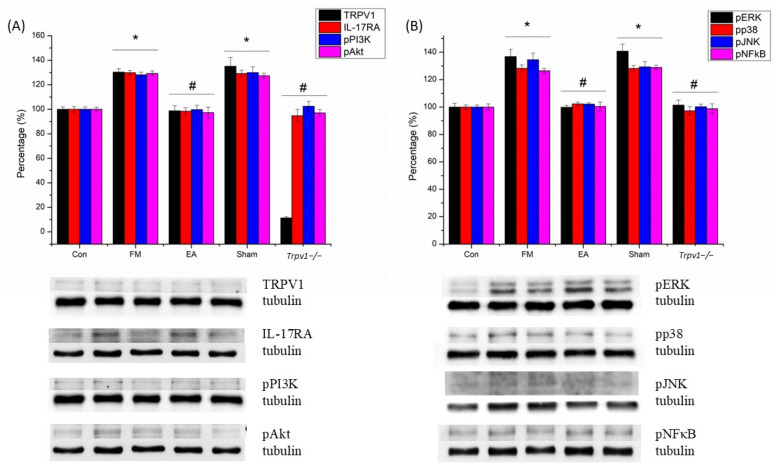
(**A**,**B**) Western blot showed that ICS increased the level of TRPV1, IL-17RA, pPI3K, pAkt, pERK, pp38, pJNK, and pNF-κB in the CB6 of mice. CB6 = cerebellum lobe VI. CON = control. FM = intermittent cold stress (ICS)-induced FM-like mice model. EA = electroacupuncture. Sham = sham EA. *Trpv1*^−/−^ = transient receptor potential vanilloid 1 gene knock out. TRPV1 = transient receptor potential vanilloid 1. IL-17RA = interleukin-17 receptor A. pPI3K = phosphorylated phosphoinositide 3-kinase. pAkt = phosphorylated protein kinase B. pERK = phosphorylated extracellular signal-regulated kinase. pp38 = phosphorylated p38. pJNK = phosphorylated c-Jun N-terminal kinase. pNF-κB = phosphorylated nuclear factor-κB. Asterisks (*) denote statistical significance compared to the control group, while hashtag symbols (#) indicate statistical significance compared to the FM group.

**Figure 9:**
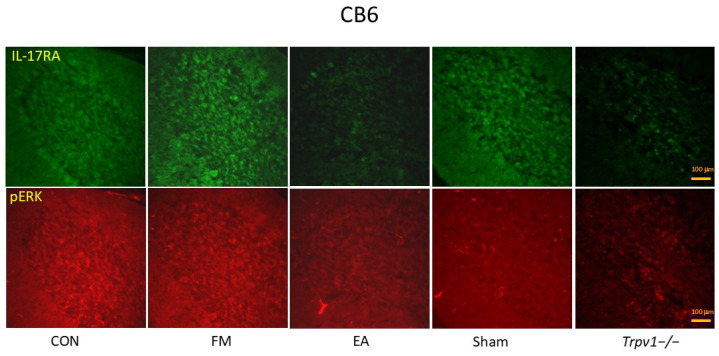
Immunofluorescence staining (n = 2) showed the increased signals of IL-17RA and pERK in FM groups. The EA and TRPV1 deletion could reverse these effects. CB6 = cerebellum lobe VI. CON = control. FM = intermittent cold stress (ICS)-induced FM-like mice model. EA = electroacupuncture. Sham = sham EA. *Trpv1*^−/−^ = transient receptor potential vanilloid 1 gene knock out. IL-17RA = interleukin-17 receptor A. pERK = phosphorylated extracellular signal-regulated kinase.

We also found that pTRPV1, pIL-17, pPI3K, pAKT, pJNK, pERK, pp38, and pNF-kB increased significantly in the CB6 of FM and Sham group mice (Figure 8; n = 6, * *p* < 0.05). The phenomenon was also abolished in the EA and *Trpv1*^−/−^ groups (n = 6, # *p* < 0.05). Immunofluorescence signals of IL-17 and ERK were low in normal mice but augmented in FM mice and were attenuated by EA but not sham EA (Figure 9; n = 2).

### 3.5. Electroacupuncture (EA) and TRPV1 Deletion (Trpv1^−/−^) but Not Sham EA Reduced IL-17-Related Signaling Pathways in the Cerebellum Lobe VII (CB7) of Mice

**Figure 10:**
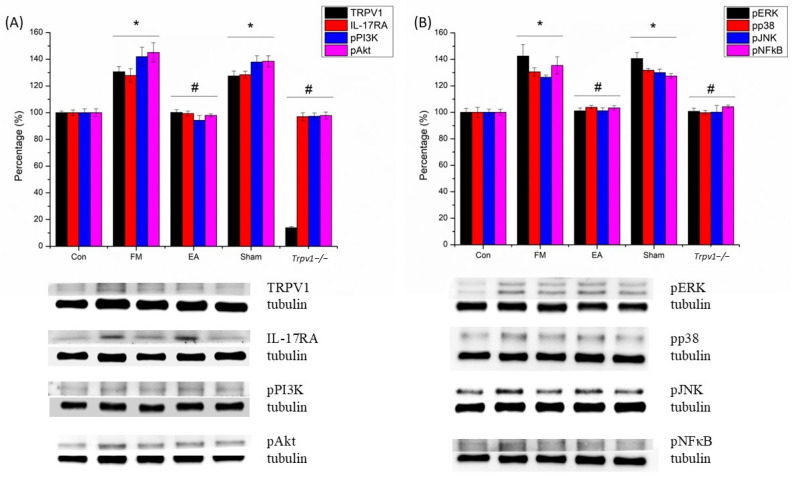
(**A**,**B**) Western blot showed that ICS increased the level of TRPV1, IL-17RA, pPI3K, pAkt, pERK, pp38, pJNK, and pNF-κB in the CB7 of mice. CB7 = cerebellum lobe VII. CON = control. FM = intermittent cold stress (ICS)-induced FM-like mice model. EA = electroacupuncture. Sham = sham EA. *Trpv1*^−/−^ = transient receptor potential vanilloid 1 gene knock out. TRPV1 = transient receptor potential vanilloid 1. IL-17RA = interleukin-17 receptor A. pPI3K = phosphorylated phosphoinositide 3-kinase. pAkt = phosphorylated protein kinase B. pERK = phosphorylated extracellular signal-regulated kinase. pp38 = phosphorylated p38. pJNK = phosphorylated c-Jun N-terminal kinase. pNF-κB = phosphorylated nuclear factor-κB. Asterisks (*) denote statistical significance compared to the control group, while hashtag symbols (#) indicate statistical significance compared to the FM group.

**Figure 11:**
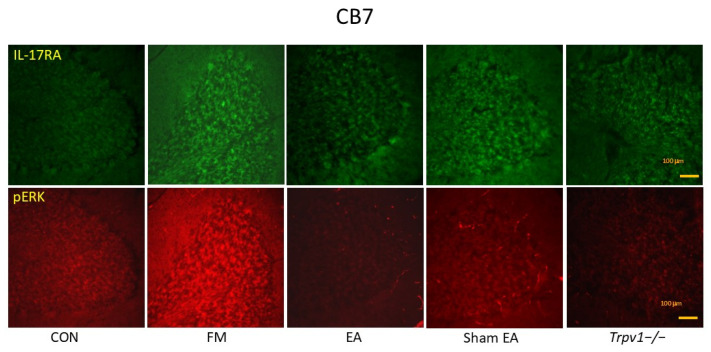
Immunofluorescence staining (n = 2) showed the increased signals of IL-17RA and pERK in FM groups. The EA and TRPV1 deletion could reverse these effects. CB7 = cerebellum lobe VII. CON = control. FM = intermittent cold stress (ICS)-induced FM-like mice model. EA = electroacupuncture. Sham = sham EA. *Trpv1*^−/−^ = transient receptor potential vanilloid 1 gene knock out. IL-17RA = interleukin-17 receptor A. pERK = phosphorylated extracellular signal-regulated kinase.

The contents of pTRPV1, pIL-17, pPI3K, pAKT, pJNK, pERK, pp38, and pNF-kB were elevated in the CB7 of FM and Sham group mice (Figure 10; n = 6, * *p* < 0.05). Additionally, these levels markedly declined in the EA and *Trpv1*^−/−^ groups (n = 6, # *p* < 0.05). Immunofluorescence signal levels of IL-17 and ERK were amplified in FM mice when compared to normal mice (Figure 11; n = 2) and were suppressed by EA but not sham EA.

## 4. Discussion

The pathophysiological factors contributing to FM pain remain unknown. We utilized the ICS mice model with female-predominant mechanical allodynia and thermal hyperalgesia, mimicking the clinical features in the FM pathophysiology [72,73,75,76]. Confirming the results of our previous studies [16,67], ICS-induced chronic generalized pain [71] in this study was attenuated by EA at the ST36 acupoint and TRPV1 deletion (*Trpv1*^−/−^). We also found that ICS increased the plasma pro-inflammatory mediators such as IL-6, IL-17, TNFα, and INFγ, which were decreased by EA and *Trpv1*^−/−^. We also observed that EA and *Trpv1*^−/−^ but not sham EA reversed the TRPV1, IL-17RA, pPI3K, pAkt, pERK, pp38, pJNK, and pNF-κB elevated by ICS in the SSC, CB5, CB6, and CB7 of mice.

Cold stress can influence the CNS of humans [77] and cause pathological alterations in the mouse brain [78]. Neuroinflammation as a contributor to FM has been illustrated in recent studies. Accumulating evidence demonstrated that neuroinflammation in the PNS and CNS provokes central sensitization [79]. Central sensitization and abnormal peripheral input have also been implicated as key factors in FM-associated pain [80,81]. The activated pain-related neural processes are modulated by numerous neural networks involving cytokines across the peripheral tissues, spinal cord, and brain [82].

IL-17 plays a crucial role in the development of inflammatory and autoimmune diseases. It has been utilized as a therapeutic target for neuroinflammation [83] and chronic pain [84]. Additionally, elevated plasma levels of IL-17A were observed in FM patients [13,14,15].

Recent compelling evidence has demonstrated that the levels of IL-17 and IL-17Rs have increased in models of neuropathic, cancer, and inflammatory pain [84]. Moreover, drugs targeting IL-17–IL-17Rs have been shown to relieve various autoimmune diseases and alleviate chronic pain in clinical trials [84]. Our previous studies also revealed that IL-17A was elevated in the plasma of the ICS mice model [16,85,86]. In this study, we further indicated that ICS induced not only IL-17A in the plasma but also IL-17RA in the SSC, CB5, CB6, and CB7.

The importance of TRPV1 in inflammatory responses has been demonstrated. TRPV1 is expressed in peripheral structures, spinal cord, and brain for pain processing [87]. It is involved in thermal sensations and pain, as well as inflammation and immunity [88]. It was found to be expressed in immune cells, including macrophages, NK cells, dendritic cells, and T lymphocytes [89,90,91,92,93]. It was also indicated that the cutaneous TRPV1+ neurons directly perceive noxious stimuli to trigger the local type 17 immune response [44]. Moreover, TRPV1 is critical for IL-17A-induced nociceptor activation and mechanical pain in female mice. Recent reports indicate that the IL-23–IL-17A–TRPV1 axis regulates female-specific mechanical pain via neuro-immune interactions [94]. Our previous studies also demonstrated that TRPV1 signaling pathways associated with ICS-induced pain were involved in the hippocampus, medial prefrontal cortex (mPFC), PAG, SSC, thalamus (THA), CB, and anterior cingulate cortex (ACC) of mice [16,65,66,95]. However, FM patients exhibited hyper-perfusion in the SSC [96] and had more dense connections in the CB [97] when compared to healthy controls. Therefore, we extracted the proteins from the SSC and cerebellar lobules V, VI, and VII (CB5, CB6, and CB7, respectively) for analysis. In this study, *Trpv1*^−/−^ abolished the ICS-induced allodynia and hyperalgesia. *Trpv1*^−/−^ also reversed the elevated IL-17A contents in the plasma and IL-17RA in the SSC, CB5, CB6, and CB7 in the ICS mice model. This result indicated that TRPV1 was necessary for activating IL-17 not only in the peripheral tissue but also in the mouse brain.

PI3K and its downstream molecule, Akt, were identified to be involved in the expression of central sensitization following noxious inflammatory stimuli [98]. They also play important roles in the development and maintenance of chronic pain [99]. The activation of the PI3K/Akt signaling pathway and the augmentation of TRPV1 expression are induced by cold stress [78]. The mechanical and thermal hyperalgesia caused by nerve injury, incision, or inflammation can be caused by this activation [100,101,102].

MAPKs are vital for transmitting signals within cells and are key players in regulating neural plasticity and inflammatory responses. Growing evidence suggests that the three MAPK pathways (ERK, p38, and JNK) each contribute to pain sensitization following tissue and nerve injury through unique molecular and cellular mechanisms. The activation of MAPKs in neurons is crucial for initiating and sustaining neural plasticity, including both peripheral and central sensitization [103,104,105]. NF-κB is a transcription factor that regulates genes associated with inflammation and pain. Chronic pain in humans and pain resulting from inflammation and nerve damage in animals are linked to an increase in NF-B activity in immune and nervous system cells [42].

TRPV1 activation initiates downstream signal pathways, including PI3K/Akt and MAPKs, which leads to NF-κB activation within the nucleus and the transcription of target genes [106]. Additionally, IL-17A–IL-17R enhanced the PI3K/Akt and MAPK pathways as well as the downstream NF-κB [107,108,109]. Our study found that the improved IL-17A–IL-17R, PI3K, Akt, ERK, p38, JNK, and NF-κB induced in the SSC, CB5, CB6, and CB7 of FM mice needed the co-expression of TRPV1.

The effects of EA vary based on the stimulation times and frequencies. Different frequencies of EA provide distinct therapeutic benefits for various diseases. It was also indicated that the inflammatory pain models prefer their optimal frequency [110]. It is more effective in relieving sensory inflammatory pain at a frequency of 2–10 Hz than at 100 Hz and more effective in suppressing inflammation and neuropathic pain [111]. The clinical trial showed that 2 Hz EA for 20 min was effective for reducing clinical pain in FM patients [63]. Moreover, based on our previous studies, 2 Hz EA for 20 min significantly attenuated the mechanical and thermal pain-like behaviors in ICS-induced FM model mice (C57BL/6) [16]. We found that EA at ST36 attenuated ICS-induced FM-pain-like behaviors through TRPV1 and its downstream pathways in the mice’s hypothalamus, hippocampus, mPFC, PAG, and CB. This study further illustrated that EA at ST36 reduced the elevated pro-inflammatory cytokines such as IL-17, IL-6, IFNγ, and TNF-α in plasma. Moreover, EA reversed the elevated TRPV1, IL-17RA, pPI3K, pAkt, pERK, pp38, pJNK, and pNF-κB in mice’s SSC, CB5, CB6, and CB7.

## 5. Conclusions

**Figure 12:**
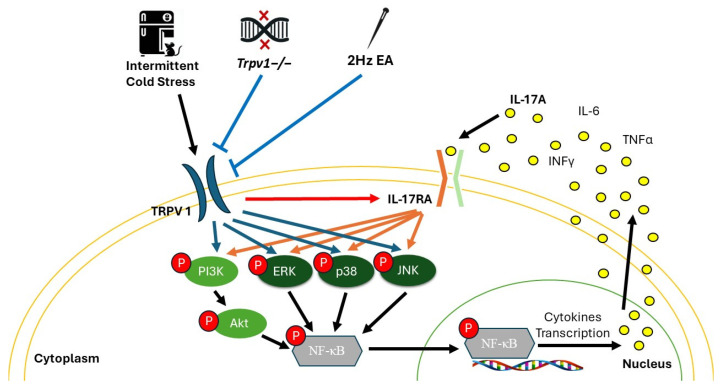
Intermittent cold stress (ICS) induced the expression of TRPV1, IL-17RA, and their downstream molecules such as pPI3K, pAkt, pERK, pp38, pJNK, and pNF-κB in the brain of mice. EA and *Trpv1*^−/−^ reversed these effects. IL-17RA and the downstream molecules could not be activated without TRPV1.

We found that FM-pain-like behavior induced in the ICS mice model elevated the plasma IL-17A and the brain IL-17RA. The results indicated that IL-17A plays a critical role in peripheral and central sensitization as well as neuroinflammation in the ICS FM mice model. Additionally, both EA and *Trpv1*^−/−^ attenuated the plasma IL-17A and the brain IL-17RA. We also found that *Trpv1*^−/−^ abolished the IL-17A, IL-17RA, and their downstream pathways, such as PI3K/Akt, MAPKs, and NF-κB. Therefore, IL-17A–IL-17RA might not induce neuroinflammation without TRPV1. Finally, our results indicated that EA relieved the ICS-induced FM mechanical and thermal pain-like behaviors maybe through the TRPV1–IL-17-related pathways in mice. It suggests that EA could lower the use of drugs and other additional pain treatment and is a potential therapeutic strategy in FM. Clinical trials should investigate the role of IL-17 in EA analgesia for FM in the future. A summary illustration of our findings is presented in Figure 12.

## 6. Limitations

There were some limitations in this study. First, a sex bias existed in this study. We chose female mice to mimic the female predominance of clinical patients. However, we do not know whether the results in male mice are like or different from those in female mice. In addition, there may be other molecules or pathways involved in EA for relieving pain in FM that need to be confirmed in the future. Third, pain is a subjective feeling, and we cannot communicate with mice. Therefore, we used the term “pain-like behavior” instead of “pain” in our results. However, we kept the “pain” description when it was from the cited articles. Finally, it is still necessary to perform study to confirm whether EA has the same effects and mechanism in humans.

## Data Availability

The original contributions presented in the study are included in the article, further inquiries can be directed to the corresponding author.
