# Correction: Yeh et al. Electroacupuncture Reduced Fibromyalgia-Pain-like Behavior through Inactivating Transient Receptor Potential V1 and Interleukin-17 in Intermittent Cold Stress Mice Model. *Brain Sci.* 2024, *14*, 869

**DOI:** 10.3390/brainsci15050529

**Published:** 2025-05-20

**Authors:** Yu-An Yeh, Hsien-Yin Liao, I-Han Hsiao, Hsin-Cheng Hsu, Yi-Wen Lin

**Affiliations:** 1 Graduate Institute of Acupuncture Science, College of Chinese Medicine, China Medical University, Taichung 404328, Taiwan; 024870@tool.caaumed.org.tw; 2 Department of Chinese Traumatology Medicine, China Medical University Hospital, Taichung 404327, Taiwan; 3 School of Post-Baccalaureate Chinese Medicine, College of Chinese Medicine, China Medical University, Taichung 404328, Taiwan; 017215@tool.caaumed.org.tw (H.-Y.L.); 002762@tool.caaumed.org.tw (H.-C.H.); 4 School of Medicine, College of Medicine, China Medical University, Taichung 404328, Taiwan; 018309@tool.caaumed.org.tw; 5 Department of Traditional Chinese Medicine, China Medical University Hsinchu Hospital, Hsinchu 302056, Taiwan; 6 Chinese Medicine Research Center, China Medical University, Taichung 404328, Taiwan

## Error in Figure

In the original publication [1], there was a mistake in the published Figure 7. **The image of Figure 9 was mistakenly used as Figure 7, leading to the duplication of Figures 7 and 9**. The corrected **Figure 7** appears below. The authors state that the scientific conclusions are unaffected. This correction was approved by the Academic Editor. The original publication has also been updated.

## Figures and Tables

**Figure 7:**
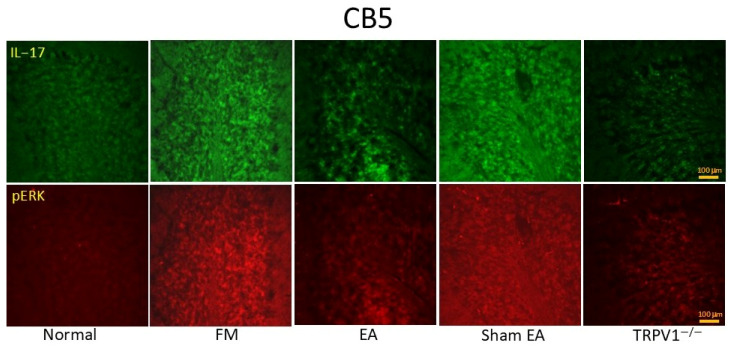
Immunofluorescence staining (n = 2) showing the increased signals of IL-17RA and pERK in the FM groups. EA and TRPV1 deletion could reverse these effects. CB5 = cerebellum lobe V. CON = control. FM = intermittent cold stress (ICS)-induced FM-like mice model. EA = electroacupuncture. Sham = sham EA. *Trpv1^−^^/^^−^* = transient receptor potential vanilloid 1 gene knock out. IL-17RA = interleukin-17 receptor A. pERK = phosphorylated extracellular signal-regulated kinase.

## References

[1] Yeh Y.-A., Liao H.-Y., Hsiao I.-H., Hsu H.-C., Lin Y.-W. (2024). Electroacupuncture Reduced Fibromyalgia-Pain-like Behavior through Inactivating Transient Receptor Potential V1 and Interleukin-17 in Intermittent Cold Stress Mice Model. Brain Sci..

